# Neutrophil-driven immunosuppression in cancer

**DOI:** 10.1016/j.cellin.2026.100356

**Published:** 2026-08-24

**Authors:** Chaoxiong Wang, Haoyu Yu, Ke Wu, Yun Zhao

**Affiliations:** a School of Basic Medical Sciences, Inner Mongolia Medical University, Hohhot, Inner Mongolia, 010059, China; b School of Life Science and Technology, ShanghaiTech University, Shanghai, 201210, China; c Key Laboratory of Multi-Cell Systems, Shanghai Institute of Biochemistry and Cell Biology, Center for Excellence in Molecular Cell Science, Chinese Academy of Sciences, University of Chinese Academy of Sciences, Shanghai, 200031, China; d School of Life Science, Hangzhou Institute for Advanced Study, University of Chinese Academy of Sciences, Hangzhou, Zhejiang, 310024, China

## Abstract

Circulating neutrophils are the most abundant leukocyte subset, yet their role in tumor progression is far more intricate than previously assumed. Neutrophils were historically viewed as short-lived effectors of innate immunity—mobilized transiently during acute inflammation and deemed largely irrelevant to chronic tumorigenesis—but this simplistic perspective has been upended by mounting evidence revealing them as a profoundly heterogeneous population. Distinct subsets can exert diametrically opposed effects, either constraining or fueling malignant growth. Within the tumor microenvironment (TME), specific neutrophil populations acquire potent immunosuppressive traits that not only shield tumors from immune surveillance but also undermine the efficacy of checkpoint blockade therapies. In this review, we trace the developmental origins of these suppressive neutrophils and draw upon recent single-cell transcriptomic studies to delineate their striking phenotypic diversity. We further dissect the molecular circuitry through which they suppress anti-tumor immunity, explore whether neutrophil-derived gene signatures can serve as reliable predictors of response to immunotherapy, and evaluate emerging therapeutic strategies. These approaches aim to block neutrophil recruitment, reprogram their functional polarization, or neutralize their suppressive activity in combination with checkpoint inhibitors. Deciphering the cues that drive neutrophils toward an immunosuppressive state will be pivotal in designing next-generation immunotherapy regimens capable of overcoming neutrophil-mediated resistance across a broad spectrum of cancers.

## 1 Introduction

The tumor microenvironment (TME) functions as a complex ecosystem wherein malignant cells co-opt host immunity to foster growth, invasion, and metastasis (Barry et al., 2023; Hedrick & Malanchi, 2022). Within this landscape, myeloid cells have emerged as pivotal orchestrators of immunosuppression and therapeutic failure (Veglia et al., 2021b). Once dismissed as short-lived, terminally differentiated effectors primarily tasked with fighting infection, neutrophils have undergone a radical conceptual revision in cancer biology. Accumulating evidence now establishes neutrophils as a dominant constituent of tumor-infiltrating myeloid populations across numerous malignancies, where they actively shape the trajectory of anti-tumor immunity (McFarlane et al., 2021).

Under oncogenic duress, substantial subsets of neutrophils acquire immunosuppressive phenotypes (Bronte et al., 2016). These cells, often referred to as polymorphonuclear myeloid-derived suppressor cells (PMN-MDSCs) within the broader tumor-associated neutrophils (TANs) compartment, cripple T-cell proliferation and effector function, expand regulatory T-cell (Treg) pools, and construct immune-privileged niches that shield cancer cells from surveillance. Their clinical significance is underscored by robust data showing that elevated baseline neutrophil counts and neutrophil-to-lymphocyte ratios (NLR) serve as independent prognostic markers and predictors of resistance to immune checkpoint blockade (ICB) (Templeton et al., 2014; Valero et al., 2021).

Recent breakthroughs in single-cell transcriptomics and spatial profiling have unveiled a degree of neutrophil heterogeneity far exceeding previous binary models, exposing context-dependent activation programs that defy simple categorization (Ng et al., 2024; Wu et al., 2024). Coupled with mechanistic insights into the signaling pathways governing neutrophil suppression, these technical advances have catalyzed novel therapeutic opportunities (Horvath et al., 2024). It is now evident that neutrophil-driven immunosuppression represents a major barrier to the efficacy of ICB. These mechanistic insights have elevated neutrophils from passive prognostic indicators to active therapeutic targets.

Deciphering these mechanisms has spurred intensive efforts to block neutrophil recruitment, reprogram their phenotypes, or neutralize specific suppressive mediators. This review synthesizes current understanding regarding the ontogeny, diversity, and functional programming of immunosuppressive neutrophils, emphasizing emerging targeting strategies designed to synergize with modern immunotherapies. We examine how single-cell technologies have redefined neutrophil heterogeneity, how metabolic rewiring fuels their suppressive arsenal, and how rationally designed combinatorial regimens may overcome neutrophil-mediated resistance to improve clinical outcomes.

## 2 Origins and development of immunosuppressive neutrophils

**Fig. 1:**
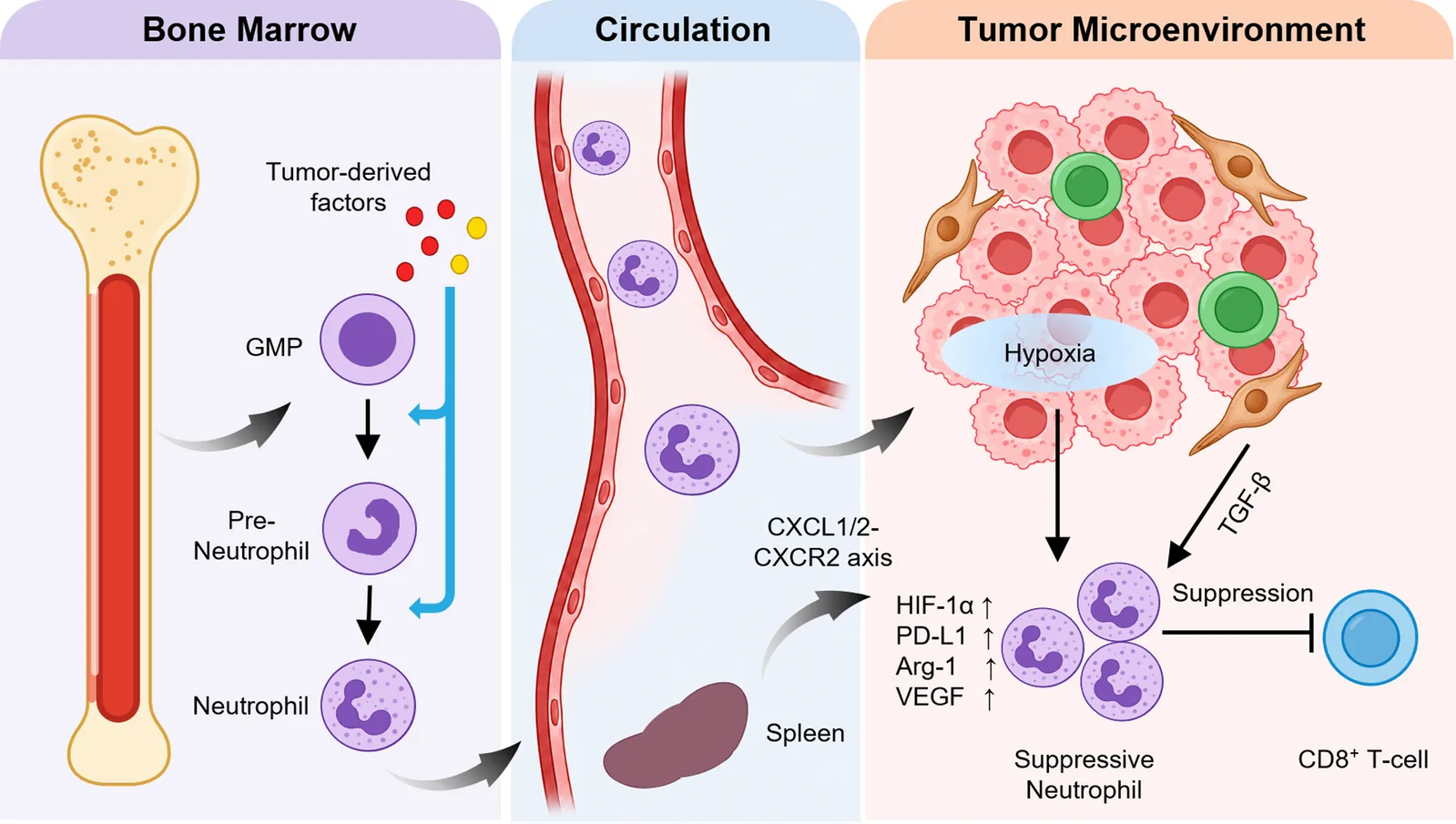
**Stepwise generation and functional reprogramming of immunosuppressive neutrophils in cancer.** Tumor-derived factors (G-CSF, GM-CSF, VEGF, IL-6) drive emergency granulopoiesis in the bone marrow, where granulocyte-monocyte progenitors (GMPs) undergo rapid expansion and epigenetic reprogramming into immature neutrophils. These cells are released into circulation, with the spleen serving as an extramedullary reservoir that amplifies the pool of suppressive neutrophils. Recruitment into the tumor bed occurs via the CXCL1/2–CXCR2 chemokine axis. Within the tumor microenvironment, local cues—principally TGF-β from tumor cells and cancer-associated fibroblasts (CAFs), together with hypoxia—enforce terminal suppressive reprogramming. This activates HIF-1α, upregulates PD-L1, arginase-1 (Arg-1), and VEGF, and ultimately paralyzes CD8^+^ T-cell anti-tumor immunity.

The emergence of immunosuppressive neutrophils in cancer represents a pathological hijacking of steady-state granulopoiesis. Systemic tumor-derived signals rewire the hematopoietic hierarchy across multiple anatomical compartments, the bone marrow, spleen, and tumor bed, establishing a continuous supply line of suppressive effectors. This section delineates the stepwise ontogeny of these cells, from progenitor commitment to terminal functional reprogramming within the TME (Fig. 1).

### 2.1 Emergency granulopoiesis and epigenetic reprogramming

Under homeostatic conditions, neutrophils develop from hematopoietic stem cells (HSCs) via a tightly regulated cascade (Evrard et al., 2018; Manz & Boettcher, 2014). Cancer disrupts this hierarchy by secreting a potent cytokine milieu, including G-CSF, GM-CSF, VEGF, and IL-6, that triggers “emergency granulopoiesis” (Sanseviero et al., 2026). This stress-adapted response prioritizes quantitative output over qualitative maturity: myeloid progenitors proliferate rapidly and are prematurely released into circulation, bypassing terminal differentiation checkpoints. The result is a systemic accumulation of immature myeloid cells. Importantly, immaturity itself appears to endow neutrophils with suppressive capacity. Analyses of human samples showed that neutrophils at the myelocyte and metamyelocyte (MC & MM) stages are intrinsically immunosuppressive, account for the majority of tumor-infiltrating neutrophils across 17 cancer types, and actively promote tumor progression (Liu et al., 2025).

Gabrilovich and colleagues characterized these cells as polymorphonuclear MDSCs (PMN-MDSCs), now recognized as the predominant immunosuppressive myeloid population in most cancers (Gabrilovich & Nagaraj, 2009). The underlying mechanism hinges on sustained STAT3 activation within myeloid progenitors, which promotes survival, enforces a suppressive lineage bias, and arrests functional maturation (Waight et al., 2013). Contemporary studies have extended this model to include epigenetic remodeling (Xu et al., 2022). Dynamic shifts in histone acetylation and DNA methylation act in concert with STAT3 to lock progenitors into a stable immunosuppressive identity during expansion. This process parallels “trained immunity”, wherein chronic exposure to tumor-derived factors reshapes the epigenetic landscape of HSCs, skewing differentiation away from protective immunity toward pathological tolerance. These epigenetic mechanisms extend beyond emergency granulopoiesis: DNA methylation reprogramming, histone modifications (acetylation, methylation, and lactylation), and ATP-dependent chromatin remodeling all help to establish and stabilize neutrophil states, and tumor-derived signals co-opt them to lock suppressive gene programs in place (Xu et al., 2022). Because such chromatin states are potentially druggable, epigenetic modulators represent a candidate avenue for therapeutically resetting pathological neutrophil identities.

### 2.2 Bone marrow imprinting and systemic conditioning

The bone marrow operates not merely as a production site but as an early training ground where suppressive traits are imprinted. Tumor-derived extracellular vesicles (EVs) and soluble factors precondition hematopoietic progenitors in situ, ensuring that newly mobilized neutrophils enter peripheral tissues pre-programmed for immunosuppression. This remote reprogramming establishes a systemic state of immune tolerance (Condamine & Gabrilovich, 2011; Du et al., 2023). By subverting myeloid differentiation pathways, tumors effectively convert the bone marrow into an active production hub for manufacturing immunosuppressive neutrophils, guaranteeing a steady cellular supply that fuels disease progression.

### 2.3 Splenic reservoirs and extramedullary hematopoiesis

Beyond the bone marrow, the spleen serves as a critical extramedullary reservoir. Under tumor burden, splenic architecture reorganizes to support extramedullary hematopoiesis, generating substantial accumulations of PMN-MDSCs (Liu et al., 2026; Wu et al., 2018). Neutrophils derived from the splenic niche contribute significantly to systemic immunosuppression. This reservoir acts as a rapid-response system, enabling tumors to escalate systemic immunosuppression during aggressive growth phases or in response to therapeutic pressure. Consequently, the spleen provides a complementary anatomical layer to bone marrow output, reinforcing neutrophil-driven immune evasion.

### 2.4 Intratumoral reprogramming

Once released from bone marrow or splenic reservoirs, circulating neutrophils are recruited into the tumor mass primarily via the CXCR2–CXCL1/CXCL2 chemokine axis (Korbecki et al., 2025). However, recruitment alone does not confer suppressive function. The terminal transformation into immunosuppressive TANs is orchestrated locally by a dense web of soluble signals, metabolic cues, and cell-contact interactions that lock these cells into a pro-tumorigenic program.

Central to this intratumoral reprogramming is TGF-β–Smad signaling. Released abundantly by tumor cells and cancer-associated fibroblasts (CAFs), TGF-β engages Smad2/3 to extinguish pro-inflammatory and cytotoxic outputs—suppressing TNF-α, ICAM-1, and ROS-mediated killing—while concurrently inducing the core suppressive toolkit, notably arginase-1 (Arg-1), MMP-9, VEGF, and PD-L1. Within this signaling milieu, neutrophils are repurposed to degrade the extracellular matrix, stimulate angiogenesis, and actively paralyze T-cell responses. Seminal work by Fridlender and colleagues demonstrated that TGF-β blockade restores anti-tumor effector functions in TANs, proving that suppressive programming is not a fixed lineage endpoint but a reversible state dictated by the microenvironment (Fridlender et al., 2009).

Concurrently, metabolic rewiring within the TME directly instructs neutrophil fate. Hypoxia stabilizes HIF-1α in infiltrating neutrophils, enhancing glycolytic flux and promoting histone lactylation events linked to the transcriptional activation of Arg-1 and other immunosuppressive gene programs (Li et al., 2026; Ugolini et al., 2025). Furthermore, lactate efflux from glycolytic tumors does more than acidify the niche; through LDHA-driven metabolic programs and HCAR1-mediated signaling, it promotes neutrophil activation and chemotactic recruitment (He et al., 2025; Wei et al., 2026).

## 3 Phenotypic diversity and functional states

Neutrophils within the tumor microenvironment display broad functional flexibility. They can directly kill malignant cells, yet they also participate actively in building an immunosuppressive niche. Early binary models laid useful groundwork, but they fall short of capturing the full complexity of neutrophil states. This section revisits the N1/N2 paradigm, examines how single-cell transcriptomics has dissected neutrophil heterogeneity across malignancies, charts the molecular signatures of immunosuppressive subsets, and clarifies the ontological relationship between PMN-MDSCs and conventional neutrophils.

### 3.1 From the N1/N2 paradigm to a continuous spectrum

Early work on TANs borrowed the M1/M2 macrophage paradigm and proposed an N1/N2 split. N1 cells were assigned anti-tumor properties, ROS production and T-cell recruitment, whereas N2 cells were linked to pro-tumor activities including angiogenesis, matrix remodeling, and immune suppression (Antuamwine et al., 2023).

The split is conceptually handy, yet it cannot accommodate the extensive phenotypic plasticity seen in both clinical and experimental settings. Neutrophil states do not fit into discrete, mutually exclusive boxes; instead, they form a continuous spectrum, with functional outputs shaped dynamically by tissue context, tumor type, disease stage, and treatment history (Hedrick & Malanchi, 2022). The discovery of PMN-MDSCs—a population that looks like mature neutrophils but potently suppresses T cells—further complicated this picture. These cells showed that shared surface markers can hide profound functional differences, making a finer classification system necessary.

This move away from fixed labels has now been formalized in an international consensus roadmap, which classifies neutrophils by developmental origin, maturation state, tissue localization, and functional program rather than by fixed subset labels (Ng et al., 2025). The framework treats neutrophil phenotypes as plastic states that are continuously remodeled by tumor progression, tissue niches, and inflammatory signals, and it calls for spatially and temporally resolved approaches—spatial transcriptomics and lineage tracing—to capture state transitions that static snapshots miss. Several studies already show the value of this view. Adoptive transfer of immature and mature neutrophils revealed that neutrophils entering pancreatic tumors, whatever their maturation stage at entry, pass through short-lived T1/T2 intermediates before converging on a long-lived, terminally reprogrammed T3 state that persists as a resident population within the tumor (Ng et al., 2024). Across organs, single-cell analyses have revealed that neutrophils acquire distinct, tissue-instructed chromatin and transcriptional programs as they enter different microenvironments (Ballesteros et al., 2020), and cross-compartment profiling defined a conserved maturation continuum (“neutrotime”) along which neutrophil phenotypes shift as cells move between bone marrow, blood, and tissues (Grieshaber-Bouyer et al., 2021). Within tumors, neutrophil states are likewise patterned by their position in the tissue, mapping onto discrete liver tumor immune microenvironment subtypes (Xue et al., 2022) and revealing lung-resident neutrophil states distinct from recruited populations (Salcher et al., 2022). Any functional classification of neutrophil states in cancer will therefore need to integrate these spatial and temporal dimensions.

### 3.2 Single-cell transcriptomics resolves neutrophil heterogeneity

Single-cell RNA sequencing (scRNA-seq) has opened a new window onto neutrophil diversity in cancer. Ng and colleagues used deterministic programming to map neutrophil states inside tumors. Their data showed that these cells undergo deterministic reprogramming in response to tumor-derived signals, rather than forming a random, heterogeneous mix (Ng et al., 2024). This work demonstrated that distinct neutrophil states can be systematically mapped and functionally defined. In parallel, large pan-cancer atlas efforts have pinpointed conserved neutrophil subsets alongside tumor-specific adaptations. In hepatocellular carcinoma (HCC), particular neutrophil clusters correlate with distinct immune microenvironment subtypes and patient prognosis (Xue et al., 2022). In pancreatic ductal adenocarcinoma (PDAC), a BHLHE40-driven neutrophil subset with hyperactive glycolysis acts as a major pro-tumor driver (Wang et al., 2023a). Work in non-small cell lung cancer has uncovered striking diversity and tissue-resident plasticity among neutrophil states at single-cell resolution (Salcher et al., 2022).

A single-cell profiling study from Wu and colleagues revealed that certain neutrophil subsets possess antigen-presenting capacity and can exert anti-tumor effects. Not every neutrophil that infiltrates a tumor is suppressive; some subpopulations clearly acquire antigen-presenting capability (Wu et al., 2024). Independent evidence now reinforces this point: Tang and colleagues identified a discrete antigen-presenting neutrophil subset that activates anti-tumor immunity and restrains the progression of non-small-cell lung cancer (Tang et al., 2025), corroborating and sharpening the antigen-presenting concept beyond a single study. These observations stress that context governs neutrophil function, and they cast doubt on the wisdom of indiscriminate neutrophil depletion.

Multi-omics approaches have added further resolution to this picture. When scRNA-seq is combined with proteomics, metabolomics, and epigenetic profiling, the data indicate that distinct metabolic and regulatory programs underpin each functional state. Pro-tumor subsets show heightened glycolytic flux and increased fatty-acid uptake. These changes meet their energy needs and, at the same time, feed suppressive function by generating immunosuppressive metabolites. By contrast, anti-tumor subsets display stronger oxidative phosphorylation and more active antigen-processing machinery, allowing them to engage productively with adaptive immune cells. Such layered molecular portraits are now guiding the design of targeted interventions that modulate specific neutrophil states without wiping out their anti-tumor potential. Some caution is warranted, however, when interpreting single-cell data on neutrophils. These cells carry little RNA, are rich in endogenous RNases, and survive poorly ex vivo, and density-gradient separation, enzymatic tissue digestion, or cryopreservation can each distort state assignments or selectively deplete fragile subsets. Telling genuine biological states apart from processing artifacts is therefore not trivial; transcriptomically defined neutrophil states are best corroborated by orthogonal spatial profiling and direct functional validation before firm mechanistic conclusions are drawn.

### 3.3 Distinct molecular signatures of suppressive neutrophils

Joint functional and transcriptomic analyses have produced solid molecular signatures that separate immunosuppressive neutrophils from their non-suppressive counterparts. In human cancer patients, the C-type lectin-like receptor LOX-1 (product of OLR1) has emerged as a defining PMN-MDSC marker. LOX-1-positive neutrophils suppress T-cell activity through an Arg-1-dependent mechanism (Condamine et al., 2016; Kim et al., 2019). Additional surface markers, CD11b, CD33, CD66b, and low or absent HLA-DR, help isolate PMN-MDSCs from peripheral blood and tumor tissue (Bronte et al., 2016).

More recent studies have widened this marker set and tied individual markers to specific immune-evasion mechanisms. CD200R marks a neutrophil subset whose autophagy is impaired; these cells expand regulatory T cells and thereby drive systemic immunosuppression (Kim et al., 2024). Similarly, CD300ld has been identified as a critical signaling hub on neutrophils for tumor-driven immune suppression; its ablation restores anti-tumor immunity and inhibits tumor growth (Wang et al., 2023c). The adaptor protein CRKL was shown to dictate anti-PD-1 resistance by mediating tumor-associated neutrophil infiltration in HCC, establishing a mechanistic link between neutrophil recruitment and immunotherapy failure (Xie et al., 2024). Furthermore, a CD10^+^ALPL^+^ neutrophil subset in HCC has been implicated in driving T-cell exhaustion, thereby conferring resistance to PD-1 blockade (Meng et al., 2023). Collectively, these findings establish that suppressive neutrophils are defined by specific, targetable molecular programs that distinguish them from conventional circulating neutrophils.

### 3.4 PMN-MDSCs: relationship to conventional neutrophils

The precise relationship between PMN-MDSCs and conventional neutrophils remains an area of active investigation. Morphologically, PMN-MDSCs are virtually indistinguishable from mature neutrophils. In mice, PMN-MDSCs are defined as CD11b^+^ Ly6G^+^ Ly6C^low^ cells, whereas in humans they are characterized as CD11b^+^ CD14^−^ CD15^+^ (or CD66b^+^) HLA-DR^low/−^ cells (Bronte et al., 2016).

One core debate centers on whether PMN-MDSCs form a separate, terminally differentiated myeloid lineage, or whether they are simply mature neutrophils that the tumor microenvironment has pathologically reprogrammed. A growing body of evidence favors the second view. Comparative analyses show that PMN-MDSCs and circulating neutrophils share a core transcriptional identity, yet they differ sharply in the expression of genes that control immunosuppressive pathways (Veglia et al., 2021a). The gain of suppressive properties seems to be a dynamic process shaped by tumor-derived cytokines, hypoxia, and metabolic stress. This implies that neutrophil suppressive activity depends on context and may be reversible. Shifting from the idea of PMN-MDSCs as a fixed lineage to viewing them as functionally plastic neutrophils carries major implications for any therapeutic strategy that seeks to modulate neutrophil-mediated immunosuppression.

This “state, not lineage” view has direct consequences for both nomenclature and therapy. If PMN-MDSCs represent a reversible functional state acquired by otherwise conventional neutrophils, classification systems built on static surface markers will inevitably misassign cells whose suppressive program is in flux; definitions that incorporate maturation stage, spatial context, and functional readouts, as advocated by recent consensus efforts (Ng et al., 2025), should therefore offer greater precision. Reversibility also matters therapeutically: suppressive neutrophils need not be eliminated but could instead be re-educated toward non-suppressive or even anti-tumor phenotypes, which favors reprogramming strategies over broad depletion.

Beyond tumor type, the organ in which a neutrophil resides imprints additional layers of functional specialization. Organ-specific cytokine landscapes, metabolic conditions, and stromal interactions shape distinct neutrophil programs: in non-small cell lung cancer, high-resolution single-cell atlases have revealed diverse tissue-resident neutrophil states that differ markedly from recruited, blood-borne populations (Salcher et al., 2022), and in hepatocellular carcinoma, neutrophil heterogeneity maps onto discrete tumor immune microenvironment subtypes (Xue et al., 2022). It is worth noting that, unlike tissue-resident macrophages—which arise embryonically and self-renew in situ—neutrophils have no dedicated, self-renewing resident compartment: all are continuously replenished from the bone marrow, and “tissue-resident” or “tissue-associated” neutrophils therefore denote marrow-derived cells that have dwelled in, and been durably imprinted by, a given tissue—an acquired functional state rather than a separate ontogenetic lineage. Distinguishing tissue-associated from newly recruited neutrophils—and understanding how local niches instruct their behavior—will be essential for generalizing mechanistic insights across cancer types.

## 4 Molecular mechanisms of immunosuppression

**Fig. 2:**
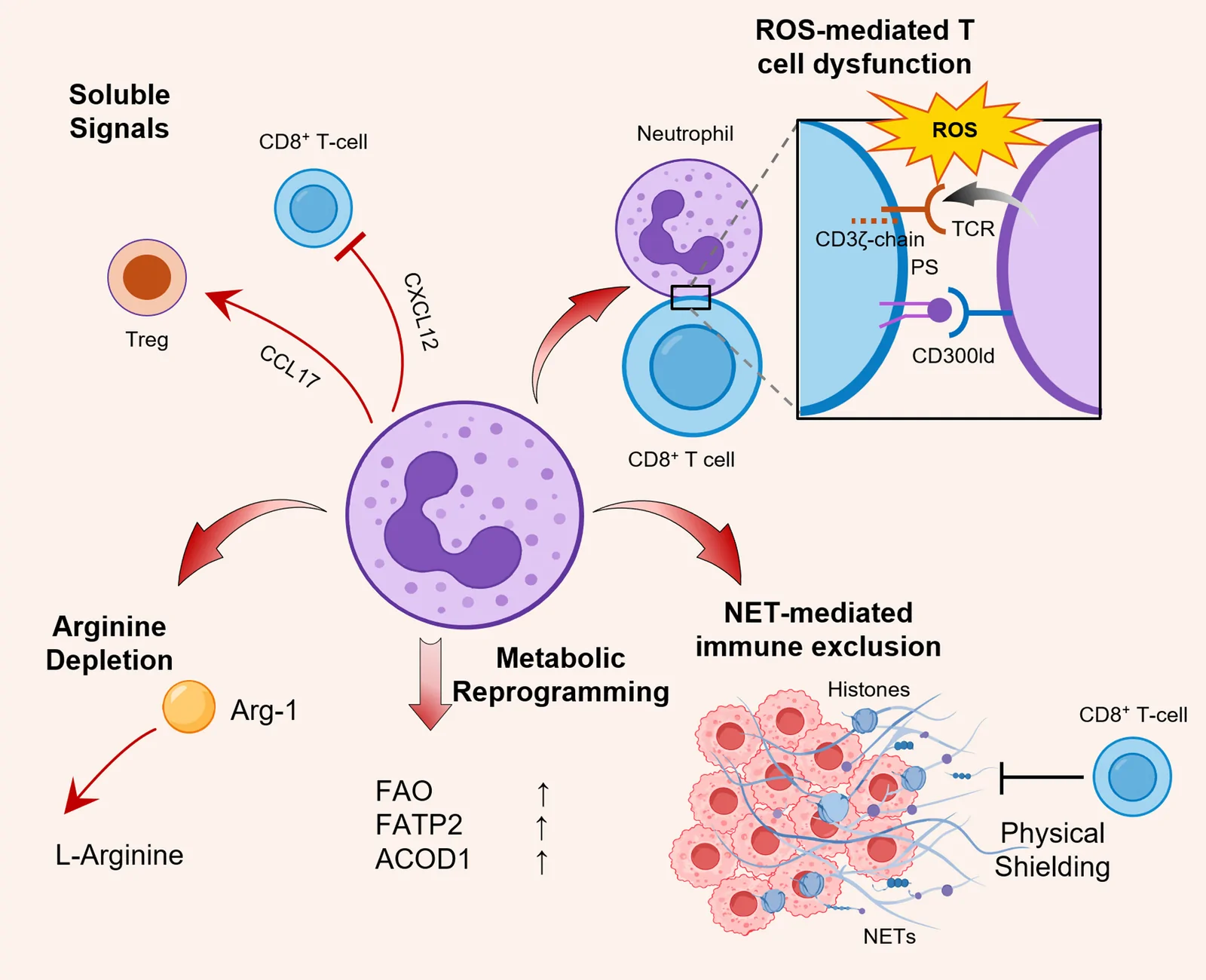
**Mechanisms of neutrophil-mediated immunosuppression in the tumor microenvironment.** Neutrophils deploy diverse strategies to disable anti-tumor immunity, including: (1) arginine depletion via arginase-1 (Arg-1); (2) ROS-mediated T-cell dysfunction through the CD300ld–phosphatidylserine (PS) axis, which tethers neutrophils to CD8^+^ T cells and disrupts TCR/CD3ζ-chain signaling; (3) NET-mediated physical immune exclusion that shields tumor cells from cytotoxic lymphocytes; (4) secretion of soluble immunoregulatory signals such as CCL17 that recruits regulatory T cells (Tregs); and (5) metabolic reprogramming characterized by enhanced fatty acid oxidation (FAO), FATP2-mediated lipid uptake, and ACOD1-dependent ferroptosis resistance. These mechanisms operate in concert to establish a robust immunosuppressive tumor microenvironment.

Neutrophils dismantle anti-tumor immunity through a confluence of coordinated molecular mechanisms. These pathways, ranging from enzymatic depletion of essential nutrients and the release of reactive species to the deployment of extracellular traps and profound metabolic reprogramming, converge to establish a robustly immunosuppressive tumor microenvironment. This section dissects these mechanisms and examines how their intersection fosters therapeutic resistance (Fig. 2).

### 4.1 Arginine depletion via arginase-1

Arginase-1 (Arg-1) stands as a canonical effector of neutrophil-mediated suppression. By catalyzing the hydrolysis of L-arginine into urea and L-ornithine, PMN-MDSCs deprive T cells of a nutrient critical for CD3ζ-chain expression, proliferation, and effector function. The resultant ζ-chain deficiency cripples antigen-specific signaling (Rodriguez et al., 2003, 2004). Concurrently, the accumulation of ornithine derivatives fuels pro-tumorigenic wound-healing and tissue-repair pathways—such as polyamine-driven cell proliferation and proline-dependent collagen synthesis—that immunosuppressive myeloid cells exploit to support tumor growth and stromal remodeling (Munder, 2009).

### 4.2 Reactive oxygen and nitrogen species

While neutrophils physiologically employ reactive oxygen species (ROS)—such as superoxide and hydrogen peroxide—via the NADPH oxidase complex for microbial killing, PMN-MDSCs repurpose this machinery to disable adaptive immunity. A prime example is peroxynitrite, formed by the reaction of nitric oxide with superoxide, which nitrates and nitrosylates TCR and CD8 molecules. This biochemical modification induces a state of functional paralysis in T cells, impairing antigen recognition and cytotoxic capacity without necessarily triggering cell death (Corzo et al., 2009; Nagaraj et al., 2007).

Given the extremely short half-life and diffusion radius of ROS, effective suppression requires intimate cellular contact (Aarts et al., 2019; Pacher et al., 2007). Recent studies have elucidated the mechanics of this interaction: the immunosuppressive receptor CD300ld, upregulated on PMN-MDSCs, binds phosphatidylserine exposed on activated CD8^+^ T cells. This interaction tethers neutrophils directly to their targets, creating the requisite proximity for ROS-mediated damage. Disruption of the CD300ld–phosphatidylserine axis dissociates these conjugates and restores anti-tumor immunity, highlighting spatial organization as a critical determinant of neutrophil suppressive function (Wang et al., 2026).

### 4.3 Neutrophil extracellular traps (NETs)

Neutrophil extracellular traps (NETs) represent a specialized form of cell death in which neutrophils release decondensed chromatin decorated with granular proteins, including histones, myeloperoxidase (MPO), neutrophil elastase (NE), and cathepsin G. Although NETs evolved as antimicrobial defenses, their aberrant formation in cancer promotes tumor progression, metastasis, and immunosuppression (Brinkmann et al., 2004; Demkow, 2021).

NETs suppress anti-tumor immunity through three complementary mechanisms. First, NET-associated Arg-1 creates localized zones of arginine depletion that inhibit T-cell function (Canè et al., 2023). Second, NETs physically shield tumor cells from cytotoxic lymphocytes, forming a barrier that prevents effective immune-tumor cell interactions (Teijeira et al., 2020). Third, NET components directly suppress T-cell proliferation and cytokine production (Song et al., 2024).

Recent work has pinned Chi3l1, a tumor-derived cytokine, as a novel trigger of NET formation in triple-negative breast cancer. Chi3l1-driven NETs weave dense extracellular matrix barriers that physically stop T cells from migrating into tumor nests, forging a clear mechanistic link between tumor-secreted factors, NET release, and immune evasion (Taifour et al., 2023). The immunosuppressive reach of NETs goes beyond physical exclusion. NET-associated chromatin complexes can directly quash T-cell receptor signaling, while histones and granular proteins push T cells toward apoptosis and dysfunction (Fang et al., 2022). Upstream of NET release, complement C5a–C5aR1 signaling induces NET formation by PMN-MDSCs and promotes metastasis (Ortiz-Espinosa et al., 2022). Given these layered roles, blocking NET formation, digesting existing traps, or neutralizing specific NET components all look like promising therapeutic avenues.

NETs also act beyond the local tumor site. Adrover and colleagues showed that tumor-elicited, vascular-restricted neutrophils release intravascular NETs that occlude the tumor microvasculature, driving a “pleomorphic” form of tumor necrosis and epithelial-to-mesenchymal transition in perinecrotic cancer cells that fuels metastatic dissemination (Adrover et al., 2025). He and colleagues, studying chronic stress, found that glucocorticoid release disrupts neutrophil circadian homeostasis and triggers NET formation, which establishes a pro-metastatic niche in the lung (He et al., 2024). These studies extend the role of NETs from local immunosuppression to systemic drivers of metastatic progression.

### 4.4 Cytokine-mediated immune regulation

Neutrophils produce a diverse repertoire of cytokines and chemokines that actively shape the immune landscape of the TME. Rather than acting as unidirectional secretors, neutrophils both release factors that reshape local immune architecture and have their own functional identity dictated by tumor- and stroma-derived cues. Through selective chemokine output, neutrophils actively recruit suppressive populations while excluding effector lymphocytes. Neutrophil-derived CCL17 attracts Tregs to tumor sites (Mishalian et al., 2014), whereas CXCL12 mediates the physical exclusion of cytotoxic T cells from tumor nests (Zboralski et al., 2017). The resulting imbalance—enrichment of regulatory cells alongside depletion of effector lymphocytes—generates a profoundly immunosuppressive milieu that erodes immunotherapy efficacy. The chemokine receptor CXCR2, highly expressed on neutrophils, serves as the principal gatekeeper of their trafficking; pharmacologic inhibition of this axis blocks pathological neutrophil accumulation in tumors and enhances immunotherapy outcomes (Steele et al., 2016).

Interleukin-8 (CXCL8) deserves special attention as a critical neutrophil chemoattractant in human cancers. Produced by both tumor and stromal cells in response to inflammatory signals, IL-8 establishes a positive feedback loop that drives neutrophil recruitment and subsequent suppression of anti-tumor immunity. Elevated serum IL-8 levels predict poor response to immune checkpoint inhibitors (Schalper et al., 2020), establishing a direct clinical link between neutrophil chemotaxis and immunotherapy resistance. Blocking the IL-8–CXCR1/CXCR2 axis represents a promising strategy to disrupt this feedforward loop and restore immunotherapy sensitivity.

Interferon signaling operates as a critical switch in this reciprocal conversation. Type I interferon–stimulated neutrophils predict favorable immunotherapy outcomes, suggesting that IFN-α/β signaling can reprogram neutrophils toward anti-tumor functional states (Benguigui et al., 2024). In stark contrast, type II interferon (IFN-γ) functions as a molecular switch that drives neutrophil PD-L1 expression, converting them into potent suppressive effectors that dampen T-cell activity and compromise treatment efficacy. Disrupting this neutrophil-intrinsic IFN-γ–PD-L1 axis restores therapeutic sensitivity, highlighting its potential as a target to overcome neutrophil-mediated resistance (Pei et al., 2026).

### 4.5 Metabolic reprogramming and lipid accumulation

The immunosuppressive behavior of neutrophils is tightly bound to their metabolic state. PMN-MDSCs display distinct metabolic traits: reliance on fatty acid oxidation (FAO) over glycolysis as a primary energy source, increased fatty acid uptake via CD36, and elevated oxidative metabolism. This metabolic rewiring is not a passive consequence of their suppressive identity; it actively drives the suppression itself. Blocking FAO-mediated lipid uptake cripples the suppressive capacity of PMN-MDSCs and unleashes anti-tumor immunity. Genetic ablation of CD36 similarly attenuates fatty acid uptake, oxidative metabolism, and immunosuppressive function in tumor-infiltrating MDSCs (Al-Khami et al., 2017; Hossain et al., 2015).

Fatty acid transport protein 2 (FATP2) has been identified as a key gatekeeper of MDSC suppressive function, controlling arachidonic acid uptake and downstream prostaglandin E2 (PGE2) synthesis. PGE2 suppresses T-cell proliferation and expands Tregs, creating a self-reinforcing cycle of immune suppression (Veglia et al., 2019). Iron metabolism has also surfaced as a critical axis. Tumor neutrophils resist ferroptosis through aconitate decarboxylase 1 (Zhao et al., 2023), which allows them to maintain suppressive activity over time; conversely, forcing these cells into ferroptosis has been proposed as a novel way to break their immunosuppressive grip. Finally, cholesterol metabolism has been implicated in neutrophil suppressive programming (Zhou et al., 2025).

## 5 Clinical relevance and biomarker applications

Translating neutrophil biology into clinical biomarkers has moved well beyond simple peripheral blood counts. Readily accessible indices such as the neutrophil-to-lymphocyte ratio (NLR) provide prognostic information, while deeper phenotypic and spatial analyses offer predictive insight into immunotherapy response. In this section we review the prognostic value of NLR, examine how specific neutrophil subsets and circulating factors predict immunotherapy outcomes, and discuss why intratumoral neutrophil density and spatial distribution are gaining importance in patient stratification.

### 5.1 Neutrophil-to-lymphocyte ratio as a prognostic indicator

The peripheral blood NLR has matured into one of the most robust and accessible biomarkers in clinical oncology. An elevated NLR serves as a composite indicator reflecting both the expansion of immunosuppressive neutrophils and the relative depletion of anti-tumor lymphocytes. It consistently correlates with poor overall survival across virtually all solid tumor types, maintaining its prognostic independence in patients receiving chemotherapy, targeted therapy, and immunotherapy. It is worth emphasizing that these associations are prognostic rather than predictive: the NLR stratifies patients by likely outcome across treatment modalities, but on its own it does not identify which patients will specifically benefit from immunotherapy—a distinction that matters for clinical trial design and for biomarker-guided treatment selection.

Specifically, within the context of ICB, a high baseline NLR consistently predicts inferior outcomes. Studies in melanoma (Capone et al., 2018), non-small cell lung cancer (NSCLC) (Kargl et al., 2019), and renal cell carcinoma (Lalani et al., 2018) have demonstrated that high neutrophil accumulation associates with lower objective response rates, shorter progression-free survival, and reduced overall survival following anti-PD-1/PD-L1 therapy. Integrating NLR with other clinical and molecular parameters into composite scores has further refined risk stratification beyond single-parameter assessments.

### 5.2 Predictive value for immunotherapy response

Beyond general prognosis, NLR and related neutrophil parameters carry specific predictive value for immunotherapy. Schalper and colleagues showed that elevated serum IL-8—a potent neutrophil chemoattractant—correlates with increased intratumoral neutrophil accumulation and diminished clinical benefit from checkpoint inhibitors, establishing IL-8 as a mechanistic biomarker that ties neutrophil recruitment to therapeutic resistance (Schalper et al., 2020). Similarly, a prospective liquid immune profile-based signature (LIPS) incorporating neutrophil counts and activation markers was developed and validated for predicting response to immune checkpoint inhibitors in recurrent and metastatic cancers (Zhou et al., 2021).

More recent studies have refined these predictions by pinpointing specific neutrophil subsets with distinct functional states. Benguigui and colleagues identified interferon-stimulated neutrophils as positive predictors of immunotherapy response (Benguigui et al., 2024), providing a functional basis for neutrophil biomarkers and suggesting that activation state, rather than absolute cell number, determines therapeutic outcome. These interferon-stimulated neutrophils display enhanced antigen presentation and reduced suppressive function, creating a microenvironment that permits T-cell-mediated tumor control. Complementary evidence comes from Gungabeesoon and colleagues, who demonstrated that an interferon-responsive, anti-tumoral neutrophil response is not merely a correlate of treatment outcome but is actively required for immunotherapy-driven tumor control—selective loss of the interferon-responsive factor IRF1 in neutrophils abolished therapeutic efficacy (Gungabeesoon et al., 2023). Together, these two studies reinforce the concept that a defined neutrophil functional state, rather than neutrophil counts per se, governs tumor responses to immunotherapy.

The predictive power of neutrophil biomarkers grows stronger when combined with tumor-intrinsic factors. Valero and colleagues demonstrated that pairing NLR with tumor mutational burden (TMB) offers complementary prognostic information for patients on checkpoint inhibitors: high-NLR/high-TMB patients fare differently from low-NLR/high-TMB patients (Valero et al., 2021). This integrative approach recognizes that neutrophil-mediated immunosuppression and tumor immunogenicity are independent yet interacting determinants of response. Refining these composite models by folding in additional neutrophil parameters—IL-8 levels, LOX-1 expression, NET markers—may enable sharper patient stratification and better treatment selection.

### 5.3 Intratumoral neutrophil infiltration and spatial distribution

Peripheral blood biomarkers are practical, but the density and spatial arrangement of neutrophils inside tumor tissue add complementary prognostic and predictive information. High intratumoral neutrophil density generally signals poor prognosis, though the relationship shifts depending on cancer type and treatment context. Spatial analyses have shown that neutrophils tend to accumulate at the invasive tumor margin and within peri-tumoral stroma, where they form a physical barrier that impedes T cells from penetrating tumor nests.

Mitra and colleagues used spatially resolved analyses to demonstrate that unfavorable neutrophil activation patterns in melanoma are tied to genomic and immune diversity (Mitra et al., 2020), linking neutrophil spatial distribution to tumor genetics and immune evasion. These spatial patterns matter when interpreting biomarkers: peripheral neutrophilia may reflect systemic immunosuppression, while neutrophil accumulation at the invasive margin directly blocks effector lymphocytes from reaching tumor cells. Taken together, peripheral blood indices and intratumoral spatial mapping offer a multi-layered framework for understanding neutrophil-mediated immunosuppression in clinical practice and for designing targeted therapeutic approaches.

## 6 Therapeutic targeting strategies

**Fig. 3:**
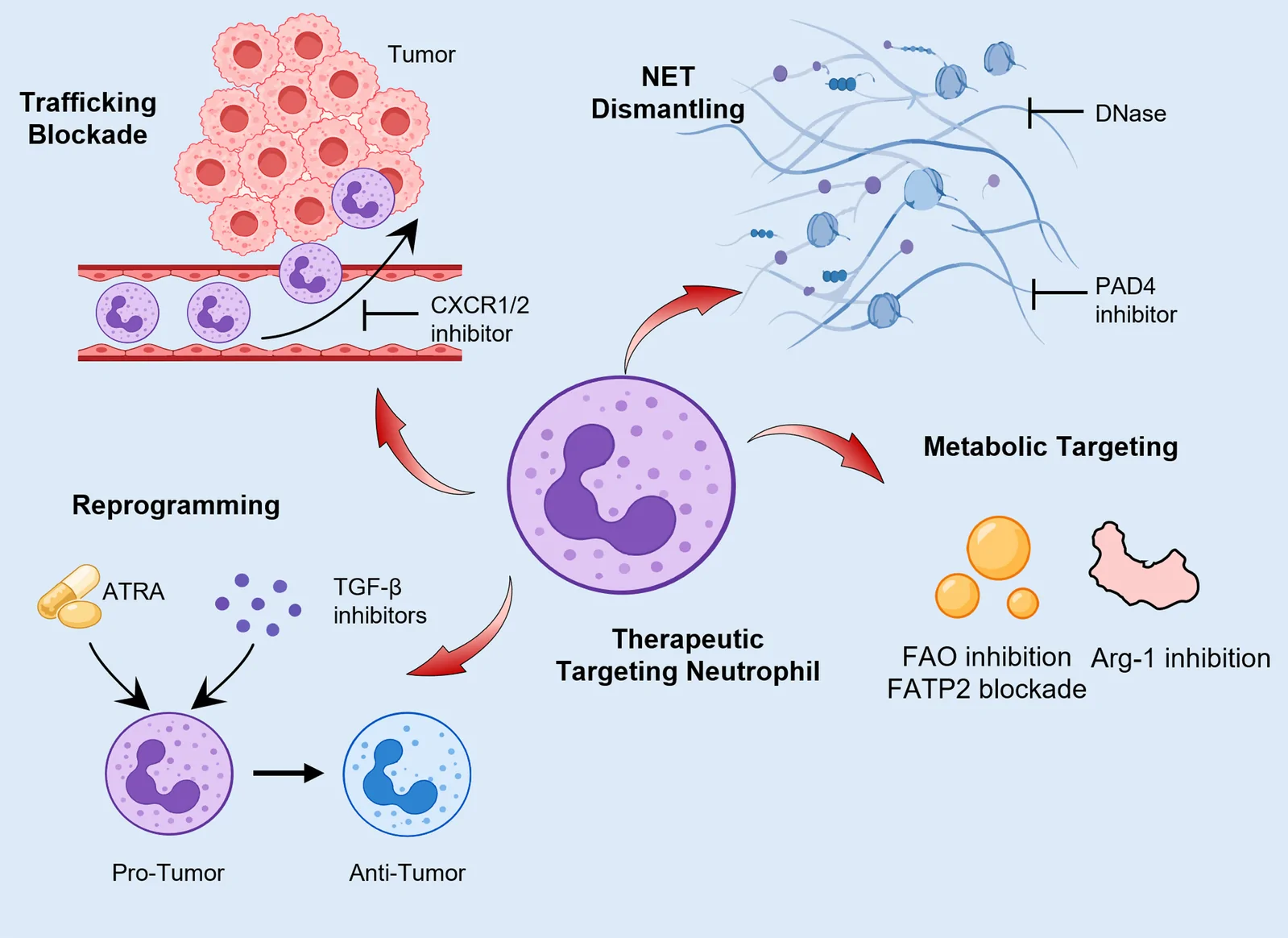
**Therapeutic strategies targeting immunosuppressive neutrophils in cancer.** Complementary approaches are highlighted: (1) trafficking blockade via CXCR1/2 inhibition to prevent neutrophil recruitment into tumors; (2) NET dismantling using DNase I to degrade pre-existing NETs, and PAD4 inhibitors to block NET formation; (3) functional reprogramming of pro-tumor neutrophils into anti-tumor phenotypes using ATRA and TGF-β pathway modulation; and (4) metabolic targeting through FAO inhibition, FATP2 blockade, and Arg-1 inhibition to impair suppressive function. These strategies collectively aim to neutralize neutrophil-mediated immunosuppression and enhance anti-tumor immunity.

Neutrophils have moved from passive bystanders to central drivers in immunosuppression, and that shift has prompted a wave of therapeutic strategies aimed at neutralizing their tumor-promoting activity. These approaches cluster along several complementary lines: blocking pathological recruitment, dismantling NETs, reprogramming suppressive cells toward anti-tumor phenotypes, exploiting unique metabolic vulnerabilities, and pairing all of the above with immune checkpoint blockade. In this section we review the mechanistic rationale and preclinical or clinical progress for each strategy, and consider how their rational combination with checkpoint inhibitors may overcome neutrophil-mediated resistance (Fig. 3).

### 6.1 Inhibition of neutrophil recruitment

Blocking neutrophil recruitment into tumors is the furthest along clinically. The CXCR1/2 axis, which steers neutrophil migration toward CXCL1, CXCL2, CXCL5, CXCL6, and CXCL8 (IL-8), has drawn the most attention. SX-682, a small-molecule CXCR1/2 inhibitor, stops MDSCs from reaching tumors and boosts NK-cell immunotherapy in head and neck cancer models (Greene et al., 2020).

Clinical translation of CXCR2 inhibition has shown promise in preclinical models. In NASH-related hepatocellular carcinoma, blocking CXCR2 makes immunotherapy work by cutting back neutrophil-driven suppression, effectively dismantling the barrier created by their accumulation in liver tumors (Leslie et al., 2022). Pancreatic cancer models tell a similar story: CXCR2 inhibition suppresses metastases and strengthens checkpoint blockade (Steele et al., 2016). The benefit probably comes from two sources—preventing fresh neutrophils from entering and tilting resident neutrophils toward less suppressive states. The catch is achieving this selectivity without stripping patients of antimicrobial defense, which will require careful dose optimization and patient selection.

### 6.2 Targeting neutrophil extracellular traps

Given the multifaceted contributions of NETs to tumor progression and immunosuppression, strategies targeting NET formation or promoting their degradation have gained considerable interest. DNase I chews up the DNA backbone of NETs and, when paired with checkpoint blockade, improves immunotherapy in preclinical models (Zhang et al., 2021). PAD4 inhibitors, which block the histone citrullination required for NET release, also restore anti-tumor immunity (Wang et al., 2023b). Researchers have also developed photoregulated enzyme delivery systems to shuttle DNase I directly into the tumor microenvironment for localized NET degradation (Chen et al., 2022). NET-targeting approaches are particularly attractive because they may preserve the beneficial antimicrobial functions of neutrophils while specifically abrogating their tumor-promoting effects.

### 6.3 Neutrophil reprogramming

Instead of wiping neutrophils out, newer approaches try to flip their functional switch. ATRA pushes immature myeloid cells, including PMN-MDSCs, toward mature neutrophils or dendritic cells with far less suppressive baggage (Nefedova et al., 2007). Clinical trials combining ATRA with checkpoint inhibitors have provided proof-of-concept that MDSC targeting can work in patients (Tobin et al., 2018, 2023).

TGF-β blockade offers another angle. Because TGF-β is a master driver of pro-tumor neutrophil polarization, shutting it down tilts neutrophils toward anti-tumor behavior, increases T-cell infiltration, and synergizes with checkpoint blockade in multiple preclinical models. Local release of TGF-β inhibitors has been shown to modulate tumor-associated neutrophils and improve pancreatic cancer response to combined irreversible electroporation and immunotherapy (Peng et al., 2022).

An emerging concept involves pharmacologically redirecting neutrophil cytotoxicity toward tumor cells, although robust preclinical validation remains limited. Activating neutrophils can convert immunosuppressive populations into tumoricidal effectors capable of direct cancer cell killing. This preserves neutrophil numbers and antimicrobial function while redirecting their cytotoxicity against tumor cells. The approach is especially attractive for combination with checkpoint blockade, potentially creating a synergistic response in which activated neutrophils kill cancer cells directly while simultaneously relieving T-cell suppression.

### 6.4 Metabolic targeting

Immunosuppressive neutrophils run on a distinct metabolic wiring that may be their Achilles' heel. PMN-MDSCs lean heavily on FAO for energy (Hossain et al., 2015); blocking this pathway impairs their suppressive capacity and enhances anti-tumor T-cell responses by restoring arginine availability and cutting ROS production. FATP2 is another metabolic chokepoint. Blocking it reduces PGE2 output by MDSCs and restores anti-tumor immunity. PGE2 suppresses T-cell proliferation and expands Tregs, so FATP2 makes an attractive target for combination immunotherapy (Veglia et al., 2019).

Arginase inhibitors, small molecules such as CB-1158, prevent arginine depletion and rescue T-cell function (Steggerda et al., 2017). NET-associated Arg-1 creates especially immunosuppressive microenvironments, and targeted neutralization of NET-bound Arg-1 enhances cancer immunotherapy efficacy (Canè et al., 2023). Cholesterol handling has recently entered the picture as well. Tumor cell-intrinsic signals rewire myeloid cholesterol metabolism to reinforce immunosuppression, and targeting this metabolic axis impairs suppressive activity (Yang et al., 2022). These metabolic interventions can be layered with checkpoint blockade: metabolic normalization removes a major barrier to T-cell function, and checkpoint inhibitors unleash the T cells that follow.

### 6.5 Rational combination with immune checkpoint blockade

The endgame for all these strategies is to make existing immunotherapies, especially checkpoint inhibitors, work better. Pairing CXCR2 blockade with anti-PD-1 has produced stronger anti-tumor effects in hepatocellular carcinoma and pancreatic cancer (Leslie et al., 2022; Steele et al., 2016). Similarly, DNase I combined with anti-PD-1 drives T-cell infiltration and tumor regression (Zhang et al., 2021). These preclinical successes establish that neutrophil-directed interventions can turn immunologically “cold” tumors “hot” and render them susceptible to checkpoint blockade.

Timing may matter as much as the drug itself. In perioperative settings, surgical trauma triggers emergency granulopoiesis and neutrophil expansion, opening a window of heightened immunosuppression that helps residual tumor cells survive and seed metastases. Targeting neutrophils during this perioperative window may therefore have disproportionate impact in preventing postoperative recurrence (Tai et al., 2018).

Small molecules are not the only option. Cell-based delivery systems are also in development to achieve tumor-specific neutrophil modulation. Neutrophil-membrane-coated nanoparticles can be loaded with therapeutic payloads and sent straight to the tumor microenvironment, capitalizing on the natural tumor-homing properties of these cells (Kang et al., 2017). CAR-neutrophil–mediated delivery of tumor-microenvironment-responsive nanodrugs has also been developed for glioblastoma chemo-immunotherapy, exploiting neutrophil infiltrative capacity to ferry drugs across the blood–brain barrier (Chang et al., 2023). These delivery systems solve the problem of achieving therapeutic concentrations inside tumors while keeping systemic toxicity low, and they represent promising platforms for integrating neutrophil-targeted agents with checkpoint blockade.

## 7 Challenges and future directions

Preclinical data look encouraging, but moving neutrophil-targeted therapies into the clinic faces stiff biological and technical headwinds. The biggest problem is selectivity: PMN-MDSCs and ordinary neutrophils share so many markers that broad depletion risks leaving patients vulnerable to bacterial and fungal infections. This necessitates careful monitoring and prophylactic antimicrobial strategies in clinical settings. Neutrophil subsets also vary wildly across cancer types and even within the same tumor, so any optimal strategy in one malignancy may flop or backfire in another. That heterogeneity demands personalized approaches guided by comprehensive biomarker profiling. Safety deserves equal weight here. Neutrophils remain indispensable for antimicrobial defense and tissue homeostasis, so systemically blocking their recruitment or activity carries a genuine risk of infectious complications and impaired tissue repair, and the therapeutic window for neutrophil-targeting agents may well be narrower than preclinical models suggest. Improving selectivity is therefore a central translational goal. Promising directions include targeting neutrophil states that are specifically enriched in tumors or other disease contexts, interfering with disease-associated pathways rather than core effector functions, and localized interventions, intratumoral delivery or neutrophil-membrane-based drug carriers, that confine activity to the tumor bed (Kang et al., 2017; Sanseviero et al., 2026).

Neutrophil plasticity adds another layer of difficulty. These cells can flip between functional states quickly in response to microenvironmental cues, so an intervention aimed at one subset may be circumvented by phenotypic adaptation. This argues for combination strategies that hit multiple functional programs simultaneously, or that fundamentally alter the microenvironmental signals driving suppressive polarization.

Biology is not the only obstacle. Neutrophils live for only a day or two in circulation, so therapy must be sustained to achieve durable effects. This poses serious pharmacokinetic and formulation challenges. Unlike long-lived lymphocytes, circulating neutrophils turn over rapidly, requiring frequent dosing, sustained-release platforms, or cell-based delivery systems to keep drug concentrations high enough.

Single-cell tools will help cut through this complexity by mapping neutrophil diversity with finer resolution and revealing new therapeutic targets (Cerezo-Wallis et al., 2026). Merging single-cell transcriptomics with spatial profiling and functional assays should yield a comprehensive atlas of neutrophil states in cancer, enabling more precise therapeutic stratification. Concurrently, advances in drug delivery, including neutrophil-membrane-coated nanoparticles and neutrophil hitchhiking strategies, offer innovative ways to modulate neutrophil function inside the tumor microenvironment while minimizing systemic toxicity. As discussed in Section 3.2, these single-cell readouts must still be weighed against the technical limitations of neutrophil profiling and confirmed by spatial and functional assays before they inform therapeutic hypotheses.

Going forward, neutrophil-directed therapy will likely advance on three fronts simultaneously: (1) selective inhibition of pathological neutrophil recruitment using chemokine receptor antagonists; (2) pharmacologic reprogramming of suppressive neutrophils toward anti-tumor phenotypes; and (3) combination regimens that weave neutrophil-targeted agents together with checkpoint inhibitors, adoptive cell therapies, and cancer vaccines. Ultimately, the success of these approaches will depend on a deeper understanding of the context-dependent cues that set neutrophil function and on the identification of robust predictive biomarkers to guide patient selection.

## 8 Conclusions

In cancer immunology, neutrophils have been completely reevaluated: no longer dismissed as minor players, they are now seen as major drivers of tumor immune evasion. The discovery of PMN-MDSCs as a discrete immunosuppressive population, coupled with work mapping their developmental origins, suppressive mechanisms, and validated clinical biomarkers, has cemented their status as legitimate therapeutic targets. Single-cell and multi-omics profiling has exposed far more heterogeneity and context-dependent plasticity among tumor-associated neutrophils than previously imagined. This has yielded a more nuanced picture of their functional states and opened up specific points for therapeutic intervention.

Moving these findings into the clinic is not straightforward. Neutrophils still serve essential antimicrobial functions, so any therapeutic strategy must avoid collateral damage to host defense while reining in their tumor-promoting activity. The most realistic way forward is to modulate neutrophil function and combine this with existing immunotherapies, rather than simply wiping these cells out. As the biology becomes clearer, selectively targeting immunosuppressive neutrophils is likely to become a standard part of next-generation immunotherapy regimens. This shift—from treating neutrophils as uniform innate killers to understanding them as adaptive, context-dependent regulators of anti-tumor immunity—should broaden the therapeutic landscape and bring lasting clinical benefit to patients who now see little response to current treatments.

## CRediT authorship contribution statement

**Chaoxiong Wang:** Writing – review & editing, Writing – original draft. **Haoyu Yu:** Writing – original draft. **Ke Wu:** Writing – original draft. **Yun Zhao:** Writing – review & editing, Supervision, Funding acquisition, Conceptualization.

## Declaration of competing interest

The authors declare that they have no known competing financial interests or personal relationships that could have appeared to influence the work reported in this paper.
